# Supplementary material for: MAGE-A3 is a prognostic biomarker for poor clinical outcome in cutaneous squamous cell carcinoma with perineural invasion via modulation of cell proliferation
Source: PLoS One. 2020 Nov 23;15(11):e0241551. doi: 10.1371/journal.pone.0241551 (PMC7682861; doi:10.1371/journal.pone.0241551)
Supplement: S1 Table — (DOCX) [file pone.0241551.s001.docx]

| **Patient**  **No.** | **Age** | **Gender** | **Tumor Site** | **Tumor Size (cm)** | **IHC Score** | **Differentiation** | **BWH Stage** |
| --- | --- | --- | --- | --- | --- | --- | --- |
| 1 | 86 | M | Scalp | 2 | < 20 | Mod | 2A |
| 2 | 96 | F | Forehead | 2.5 | 20 | Mod | 2A |
| 3 | 81 | M | Face | 2.5 | < 20 | Mod | 2B |
| 4 | 81 | M | Temple | 1.4 | < 20 | Mod | 2B |
| 5 | 64 | M | Temple | 3.1 | 21-49 | Mod | 2B |
| 6 | 45 | F | Forehead | 2.5 | 21-49 | Mod | 2B |
| 7 | 65 | M | Ear | 2.5 | 50-999 | Poor | 3 |
| 8 | 90 | M | Arm | 2 | >1000 | Poor | 3 |
| 9 | 88 | M | Scalp | 2 | >1000 | Poor | 3 |

**S1 Table: Clinical characteristics of patients with cSCC with PNI.**

Summary of 9 patients with cSCC with PNI, including 6 BWH stage 2 and 3 BWH stage 3. Patients are arranged in order of BWH stage. Mod = moderately differentiation. Poor = poorly differentiation.
