# Supplementary figures and images for: MAGE-A3 is a prognostic biomarker for poor clinical outcome in cutaneous squamous cell carcinoma with perineural invasion via modulation of cell proliferation
Source: PLoS One. 2020 Nov 23;15(11):e0241551. doi: 10.1371/journal.pone.0241551 (PMC7682861; doi:10.1371/journal.pone.0241551)

Figure 3B

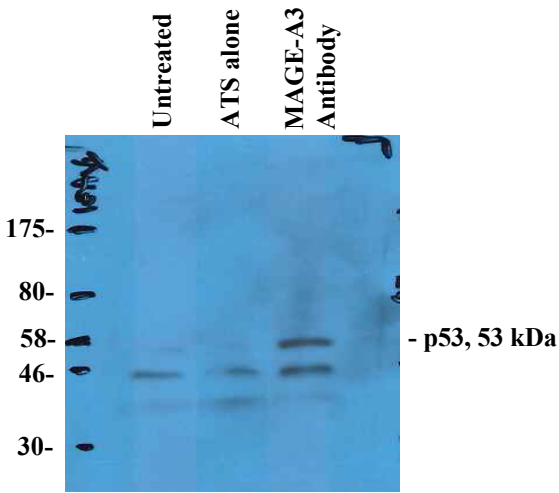

Figure 3B

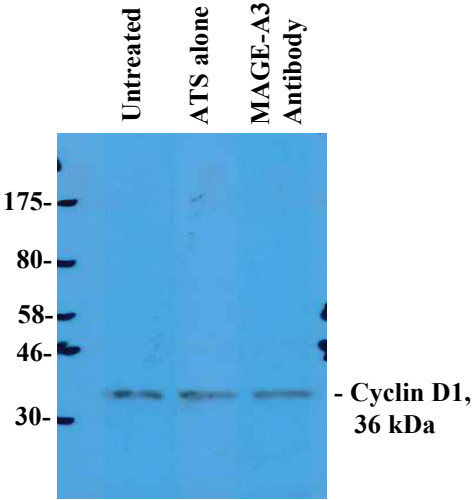

Figure 3B

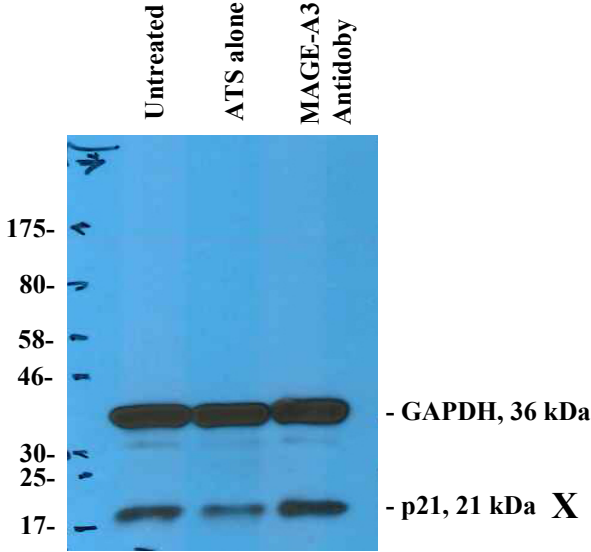

Figure 4B

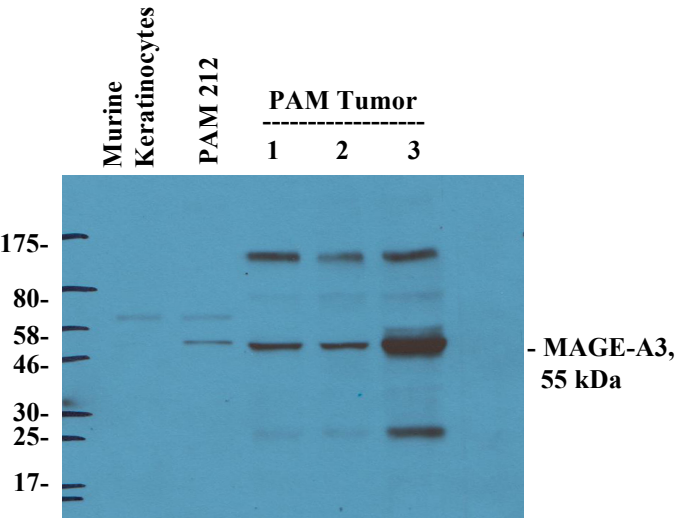

Figure 4B

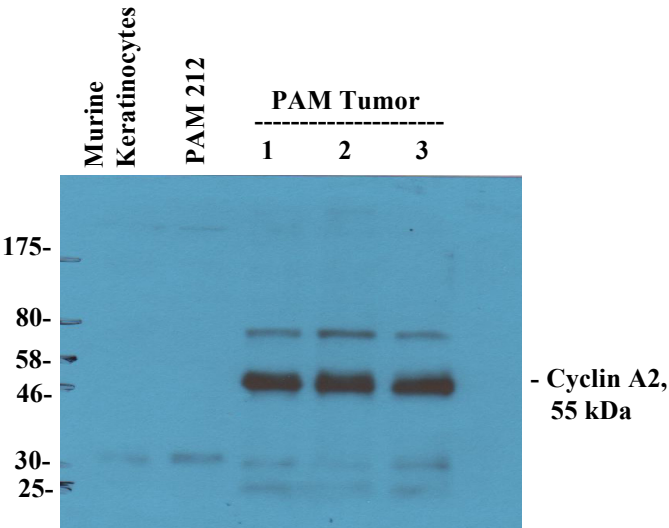

Figure 4B

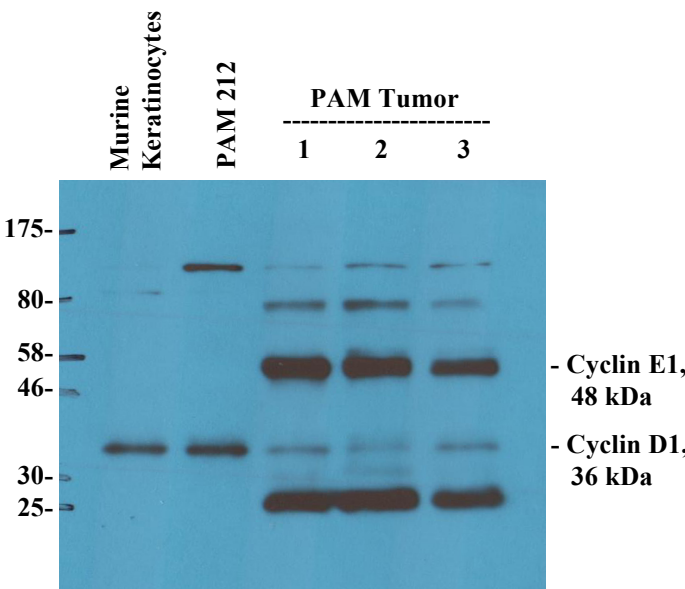

Figure 4B

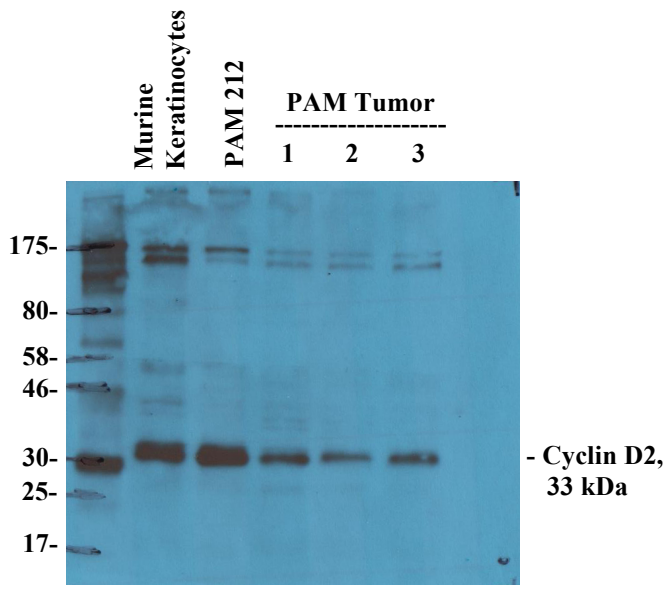

Figure 4B

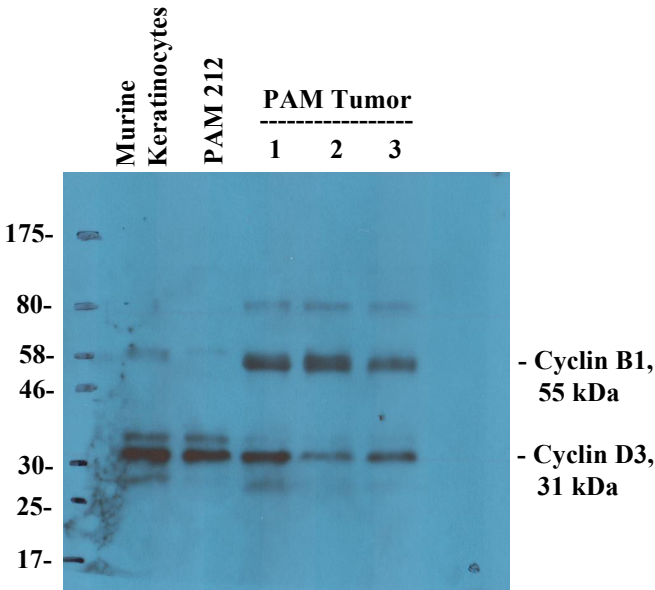

Figure 4B

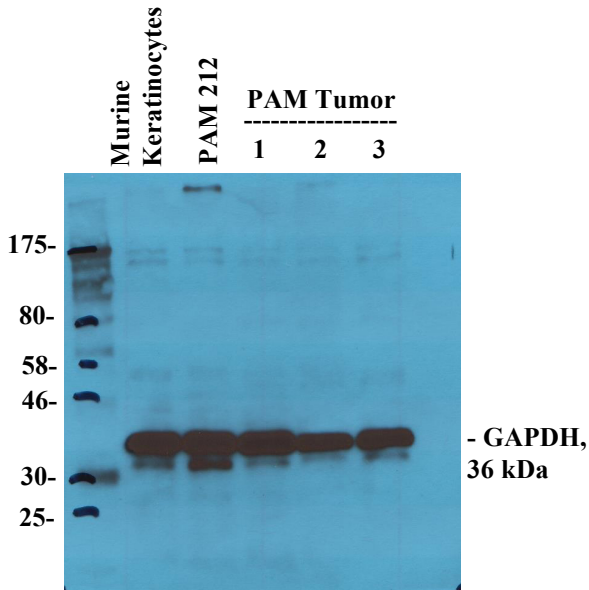

Figure 5C

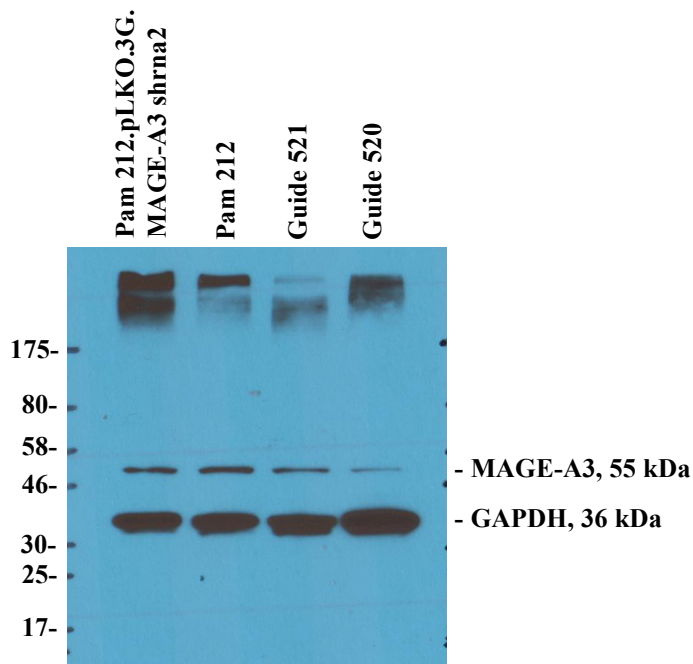

Supplement: S1 Raw images — (PDF) [file pone.0241551.s002.pdf]
