# Supplementary material for: MAGE-A3 is a prognostic biomarker for poor clinical outcome in cutaneous squamous cell carcinoma with perineural invasion via modulation of cell proliferation
Source: PLoS One. 2020 Nov 23;15(11):e0241551. doi: 10.1371/journal.pone.0241551 (PMC7682861; doi:10.1371/journal.pone.0241551)
Supplement: Attachment — Submitted filename: Response to Samantha Russell.doc [file pone.0241551.s003.doc]

September 25, 2020

RE: Manuscript Entitled: **MAGE-A3 is a prognostic biomarker for poor clinical outcome in cutaneous squamous cell carcinoma with perineural invasion via modulation of cell proliferation**

Samantha Russell

*PLOS ONE*

Dear Ms. Russell:

Thank you for your technical review of our revised manuscript*.* The revised manuscript has been substantially strengthened after addressing all the important critiques regarding technical assessments. We have included a point-by-point response to the comments below.

**Editor Comments**

Major comments:

(1) Thank you for including your ethics statement on the online submission form: " All human studies were reviewed and approved by the institutional review board at NYU Langone Medical Center. Written informed consent was obtained for all patients before their participation, and the study was performed with strict adherence to the Declaration of Helsinki Principles. Human Subjects protocol: IRB protocol 16-00122.

Animal studies described were reviewed and approved by the Institutional Animal Care and Use Committee at NYU Langone Medical Center and were conducted according to the requirements established by the American Association for Accreditation of Laboratory Animal Care. All procedures were approved by the Institutional Animal Care and Use Committee before the initiation of any studies. Animal Protocol number: IA16-00510." To help ensure that the wording of your manuscript is suitable for publication, would you please also add this statement at the beginning of the Methods section of your manuscript file.

Response: We thank you for this advice. The following information has been added to the beginning of the Methods section. “All human studies were reviewed and approved by the institutional review board at NYU Langone Medical Center. Written informed consent was obtained for all patients before their participation, and the study was performed with strict adherence to the Declaration of Helsinki Principles. Human Subjects protocol: IRB protocol 16-00122.

Animal studies described were reviewed and approved by the Institutional Animal Care and Use Committee at NYU Langone Medical Center and were conducted according to the requirements established by the American Association for Accreditation of Laboratory Animal Care. All procedures were approved by the Institutional Animal Care and Use Committee before the initiation of any studies. Animal Protocol number: IA16-00510.” This information appears on Page 5 Line 16 to Page 6 Line 5.

(2) Please provide additional information about each of the cell lines used in this work, including any quality control testing procedures (authentication, characterisation, and mycoplasma testing). For more information, please see [http://journals.plos.org/plosone/s/submission-guidelines#loc-cell-lines](http://journals.plos.org/plosone/s/submission-guidelines#loc-cell-lines).

Response: We thank you for this valuable comment. A431 cells were obtained from American Type Culture Collection (ATCC CRL-1555; Manassas, VA). Information on Pam 212 cell lines, including Pam 212 (gift from Dr. Stuart Yuspa, NIH, National Cancer Institute)(Li, Shukla et al. 2010) and Pam 212 LY2 (gift from Dr. Carter Van Waes, National Institute of Deafness and Other Communication Disorders) (Loukinova, Dong et al. 2000), has also been provided. Prior to use in experiments, all cells were routinely assessed for mycoplasma contamination with MycoAlert Mycoplasma Detection Kit (Cat # LT07-418, Lonza). All information about cell lines used in this study has been described in the Materials and Methods section under the subheading “Cell lines and cell culture” (Page 8 Line 1-12).

(3) In your Methods section, please provide additional information about the participant recruitment method and the demographic details of your participants. Please ensure you have provided sufficient details to replicate the analyses such as: a) the recruitment date range (month and year), b) a description of any inclusion/exclusion criteria that were applied to participant recruitment, c) a table of relevant demographic details, d) a description of how participants were recruited, and e) descriptions of where participants were recruited and where the research took place.

Response: We thank you for this important suggestion. IRB approval at NYU Langone Medical Center was acquired prior to the study (IRB protocol 16-00122). Surgical discard that would otherwise be disposed of as medical waste was obtained from a total of 24 patients who had no history of organ transplants and presented for dermatologic evaluation of primary cSCC at NYU Langone Medical Center. Participation involved the use of surgical discard and correlation with clinical characteristics of the tumor. Participation was voluntary. Among the participants, nine patients with primary cSCC who revealed PNI in their pathological reports were chosen for follow-up of their clinical outcomes, and their tumor specimens were analyzed for further investigation. Other molecular characteristics of these patient tumors were described in another study (Abikhair, J Invest Dermatol. 2017 Mar;137(3):775-778). This information has been added to Page 6, Lines 8-17. Demographic information is also reported in Supplemental Table S1 and presented below.

| **Patient**  **No.** | **Age** | **Gender** | **Tumor Site** | **Tumor Size (cm)** | **IHC Score** | **Differentiation** | **BWH Stage** |
| --- | --- | --- | --- | --- | --- | --- | --- |
| 1 | 86 | M | Scalp | 2 | < 20 | Mod | 2A |
| 2 | 96 | F | Forehead | 2.5 | 20 | Mod | 2A |
| 3 | 81 | M | Face | 2.5 | < 20 | Mod | 2B |
| 4 | 81 | M | Temple | 1.4 | < 20 | Mod | 2B |
| 5 | 64 | M | Temple | 3.1 | 21-49 | Mod | 2B |
| 6 | 45 | F | Forehead | 2.5 | 21-49 | Mod | 2B |
| 7 | 65 | M | Ear | 2.5 | 50-999 | Poor | 3 |
| 8 | 90 | M | Arm | 2 | >1000 | Poor | 3 |
| 9 | 88 | M | Scalp | 2 | >1000 | Poor | 3 |

(4) At this time, we ask that you please add scale bars to the microscopy images provided in Figure 1, 2 and 4 and refer to the scale bar in the corresponding Figure legend.

Response: We thank you for this important suggestion. Please note, Figures 1, 2 and 4 were modified to Figures 2, 3 and 5 in the revised manuscript that we recently submitted. All microscopic images in Figure 2 now include scale bars on the lower right corners, and each bar indicates 100 µm. Scale bar information has been added to the legend of Figure 2 on Page 17 Lines 5-6. Scale bars indicate 100 µm in Figure 3D, and this information appears in the corresponding legend on Page 19 Line 2. Scale bars are located on the lower right corners of microscopic images in Figure 5F and 5G. The information is provided in the legend of Figure 5 Page 21 Lines 11-14.

(5) In your Methods section, please include the full name of the IRB/ethics committee and IACUC that reviewed and approved these studies, including the name of the affiliated institution if applicable. We additionally ask that you include your IRB/ethics committee approval number and IACUC approval number in your ethics statement.

Response: We thank you for this valuable suggestion. This information has been added to the Materials and Methods section on Page 5 Line 16 to Page 6 Line 5. The IRB/ethics committee approval number and IACUC approval number have been added to the ethics statement upon submission.

(6) We suggest you thoroughly copyedit your manuscript for language usage, spelling and grammar. If you do not know anyone who can help you do this, you may wish to consider employing a professional scientific service.

Response: We thank you for this comment. We have carefully proofread the entire article for language usage, spelling and grammar.

We believe that we have addressed all the points raised by the editor. Please feel free to contact us if you have any additional questions or concerns. We thank you once again for this technical review. We look forward to hearing from you soon.

Sincerely,

John A. Carucci, MD, PhD
